# Semiochemicals to enhance herbivory by Diorhabda carinulata aggregations in saltcedar (Tamarix spp.) infestations

**DOI:** 10.1002/ps.4848

**Published:** 2018-02-23

**Authors:** Alexander M Gaffke, Sharlene E Sing, Tom L Dudley, Daniel W Bean, Justin A Russak, Agenor Mafra‐Neto, Paul A Grieco, Robert KD Peterson, David K Weaver

**Affiliations:** ^1^ Department of Land Resources and Environmental Sciences Montana State University Bozeman MT USA; ^2^ USDA Forest Service Rocky Mountain Research Station Bozeman MT USA; ^3^ Marine Science Institute University of California Santa Barbara CA USA; ^4^ Colorado Department of Agriculture Palisade Insectary Palisade CO USA; ^5^ Department of Chemistry and Biochemistry University of California Santa Barbara Santa Barbara CA USA; ^6^ ISCA Technologies Inc. Riverside CA USA; ^7^ Department of Chemistry and Biochemistry Montana State University Bozeman MT USA

**Keywords:** aggregation pheromone, plant volatiles, attractant, biological control, invasive weed

## Abstract

**BACKGROUND:**

Semiochemicals for monitoring, attracting or repelling pest and beneficial organisms are increasingly deployed in agricultural and forest systems for pest management. However, the use of aggregation pheromones and host‐plant attractants for the express purpose of increasing the efficacy of classical biological control agents of weeds has not been widely reported. Therefore, we conducted field‐based assays to determine if a specialized wax‐based matrix impregnated with an aggregation pheromone of the northern tamarisk beetle Diorhabda carinulata (Desbrochers) or host‐plant volatiles could increase the efficacy of D. carinulata.

**RESULTS:**

The aggregation pheromone and host‐plant volatiles were formulated for field application using a wax‐based matrix. Reported release rates suggest that this matrix is a viable formulation for enhancing D. carinulata aggregations under field conditions. Pheromone‐treated saltcedar plants (Tamarix spp.) not only had higher densities of adult and larval D. carinulata, but also sustained greater levels of foliar damage than control plants. Increased damage from the focused feeding of D. carinulata caused an increase in foliar dieback and decrease in live canopy volume of semiochemical‐treated plants.

**CONCLUSION:**

Field deployment of these semiochemical formulations could be useful in directing populations of D. carinulata for increased impact on Tamarix spp. © 2018 The Authors. *Pest Management Science* published by John Wiley & Sons Ltd on behalf of Society of Chemical Industry.

## 1 INTRODUCTION

Insect herbivory threatens plant resources of aesthetic, ecological and economic value to humans.^1^ Plant protection often relies on several tactics to attain successful pest management. One such tactic is the behavioral manipulation of pest insects. There are many examples of behavioral manipulation for the management of insect pests, especially those using attractive semiochemicals to lure pests to a targeted management area.^2^, ^3^ Use of semiochemical‐based ‘pull’ strategies in pest management can be found in diverse pest‐crop systems including Helicoverpa spp. in cotton, Sitona lineatus (Linnaeus) in beans, Delia antiqua (Meigen) in onion and Dendroctonus spp. in coniferous forests.^4^, ^5^, ^6^ A semiochemically based pull system has also been used to manipulate the density of larvae and adults, and to alter oviposition preference in Leptinotarsa decemlineata (Say).^7^ These ‘pull’ strategies commonly utilize attractive stimuli such as pheromones, host volatiles and non‐host volatiles.

The semiochemically based strategies mentioned above focus on manipulating pest species to minimize the impact of herbivory on crops or timber. One pest management strategy, classical biological control of weeds using insect agents, stands out as being exclusively reliant on insect herbivory to control target plant species.^8^ Successful biological control of weeds depends on insect herbivory that is intense enough to have a profound negative impact on the target plant at the population level.^8^, ^9^

Of related interest is whether the concept of insect behavioral manipulation, now prevalent in crop pest management, can be applied to classical weed biological control to increase the impact of agents. Martel et al. 7 demonstrated that applying synthetic host volatile compounds to potato resulted in an increase in the population density of L. decemlineata on treated plants. The study identified the potential role of semiochemicals in the spatial manipulation of herbivorous insect species, specifically to direct or guide colonization, and facilitate aggregation. This concept, if applied to classical weed biological control, could intensify herbivory on the target weed, potentially resulting in increased efficacy of the biocontrol agent.^10^

Semiochemically mediated spatial manipulation of populations of an arthropod biological control agent was evaluated using the agent–host interactions of D. carinulata and Tamarix spp. in the field. This plant–insect system was well‐suited to this investigation because aggregation‐facilitating semiochemicals for this agent have been identified and are well characterized. These include the aggregation pheromones (2E,4Z)‐2,4‐heptadien‐1‐ol (2E,4Z‐7:OH) and (2E,4Z)‐2,4‐heptadienal, and a suite of green leaf volatiles that includes (Z)‐3‐hexenal (3Z‐6:Ald), (E)‐2‐hexenal (2E‐6:Ald), (Z)‐3‐hexen‐1‐ol (3Z‐6:OH) and (Z)‐3‐hexenyl acetate (3Z‐6:OAc).^11^, ^12^ Aggregation pheromones are produced by reproductive males and attract both adult male and female D. carinulata equally.^11^ Green leaf volatiles are produced by the host plant and are induced by the feeding of D. carinulata.^12^ Similar to the aggregation pheromones, the green leaf volatiles are highly attractive to male and female D. carinulata.^12^

Tamarix species are woody trees or shrubs native to arid regions of Asia, Europe and Africa that were introduced to North America beginning in the early 1800s.^13^, ^14^ Ten species of Tamarix have been introduced to North American and have experienced extensive hybridization.^15^, ^16^, ^17^ To simplify discussion of this genus in North America, the Tamarix species and their hybrids are hereafter referred to as Tamarix. The hybrid Tamarix that occurs in North America are readily utilized by D. carinulata.^18^

A biological control program initiated in the late 1960s, primarily to manage extensive Tamarix invasion in the southwestern USA, led to the approval for field release of the northern tamarisk beetle D. carinulata.^19^, ^20^, ^21^ D. carinulata overwinters in the adult stage in the litter below Tamarix plants.^22^ Adults begin laying eggs on the Tamarix foliage within 4–7 days after spring emergence.^23^ Once the eggs hatch, the larvae progress through three stages within the Tamarix canopy.^22^ Following completion of the third stage, larvae drop from the canopy and form pupal cells in the litter.^22^ After metamorphosis, the F1 adults emerge from the soil in mid‐July. D. carinulata populations are multivoltine, but typically bivoltine in the northern part of their North American range, with the F1 adults actively reproductive and the F2 adults entering a reproductive diapause.^22^, ^24^ D. carinulata is highly mobile while reproductive and is known to disperse 25 km/year.^25^ Once adults enter reproductive diapause, dispersal behavior ceases, nutritional reserves are developed, and they burrow into the litter to overwinter, emerging the following spring to reproduce.^22^, ^23^, ^26^ Males in reproductive diapause do not produce the aggregation pheromone.^23^

All life stages of D. carinulata feed on the foliage of Tamarix by rasping and scraping the leaves and photosynthetic stems.^27^ These feeding activities damage leaf surface structures, resulting in compromised water regulation in affected plants. Impaired water regulation results in desiccation and ultimately defoliation of the plant.^28^, ^29^, ^30^ Feeding by D. carinulata can dramatically increase stress on plants, potentially reducing carbon storage, impairing leaf production and reducing root mass in the years following a defoliation event.^30^, ^31^, ^32^ Multiple years of intense herbivory by D. carinulata can result in Tamarix mortality.^31^, ^33^

Many areas where D. carinulata was released and established have not attained an appreciable level of D. carinulata‐mediated Tamarix control.^34^, ^35^ For example, releases made near Lovell, Wyoming in 2001 resulted in the establishment of D. carinulata, but impacts on Tamarix have not been widespread.^35^ In particular, control of Tamarix by D. carinulata is not evident, even though the established stands at Lovell are considered young, less robust and at a lower density than other infestations found in North America.^36^, ^37^, ^38^, ^39^ This lack of control is likely due to infrequent but recurring flooding that prevents populations of D. carinulata from expanding to the levels required to appreciably impact Tamarix.^35^ The presence of D. carinulata near Lovell, Wyoming and the lack of measurable control provide a unique opportunity to investigate the ability of semiochemicals to potentiate the impacts of D. carinulata by aggregating populations on specific Tamarix plants. In this study, we report on the use of semiochemicals to enhance aggregations and intensify D. carinulata herbivory on treated plants, and thereby increase efficacy of this agent in northern Tamarix infestations.

## 2 MATERIALS AND METHODS

### 2.1 Field experiment location and insect source

Two research locations were targeted along the Bighorn River near Lovell, Wyoming. Locations were chosen based on the existing infestation of Tamarix and the presence of D. carinulata. Naturally occurring and reproducing populations of D. carinulata have been established on this site since releases were initially made in 2001.^22^ The beetles established in this area were from a population that originated near the town of Fukang, Xinjiang, China.^19^, ^22^

### 2.2 Lure and chemicals

SPLAT® (ISCA Technologies, Riverside, CA, USA), a commercially available flowable wax‐based controlled release matrix, was used to determine the effects of aggregation and behaviorally active host‐plant semiochemicals on D. carinulata densities in the field. SPLAT was used because of its potential to maintain a constant release rate over extended periods.^40^ SPLAT has been used in both agricultural and forest applications.^41^, ^42^ Although SPLAT is commercially available, this is a novel application of SPLAT utilizing these specific semiochemicals. The alcohol component of the D. carinulata aggregation pheromone was synthesized according to Petroski.^43^ Cossé et al. 11 identified paired pheromone components but assays indicated that trap capture was the same using only the single alcohol component of the aggregation pheromone; therefore only the alcohol component was formulated in SPLAT. The aggregation‐causing green leaf volatiles 2E‐6:Ald, 3Z‐6:Ald, 3Z‐6:OH and 3Z‐6:OAc (purity >95%) were purchased from Sigma‐Aldrich (St. Louis, MO, USA), and 3Z‐6:Ald (50% triacetin) was purchased from Bedoukian Research Inc. (Danbury, CT, USA).

Three types of lures were prepared for field testing. The first consisted of SPLAT impregnated with the aggregation pheromone (hereafter referred to as PH). The second consisted of SPLAT impregnated with a blend of the four green leaf volatiles (hereafter referred to as PL). The third lure consisted of the base formulation of SPLAT with no behaviorally active ingredients added, to act as a control formulation (hereafter referred to as BL). Percent active ingredient of each semiochemical per treatment formulation was as follows: PH formulation, 2.17% 2E,4Z‐7:OH; and PL formulation, 0.85% 3Z‐6:Ald, 0.85% 2E‐6:Ald, 0.17% 3Z‐6:OH and 0.13% 3Z‐6:OAc. The BL formulation contained the SPLAT matrix only with no semiochemicals.

### 2.3 Release‐rate analysis

Eight replicates of the PH and PL lure formulations were applied to yellow cattle ear tags as 1‐g doses (dollops). SPLAT formulations, which were stored at 4 °C in a chromatography fridge while not in use, were removed from the fridge and 1‐g dollops were applied to the cattle ear tags. After application of the dollop, the cattle ear tags were transported (< 1 km) and subsequently exposed to outdoor conditions at the Bozeman Forestry Sciences Laboratory (USDA Forest Service–Rocky Mountain Research Station) on the campus of Montana State University (Bozeman, MT, USA). The dollops of SPLAT were exposed to ambient outdoor conditions for the remainder of the experiment, except for removal and transport to the laboratory for brief volatile collections. Volatile collections of the field‐aged dollops were made at 1, 3, 5, 10, 15, 25 and 31 days to determine the release rates of semiochemicals from the matrix. Collections were made in glass volatile collection chambers (internal diameter 35 mm, length 625 mm) over varying periods (15 min at 1, 3, 5 and 10 days; 30 min at 15 days; and 1 h at 25 and 35 days). Volatile collection traps containing 30 mg of super‐Q (Alltech Associates, Inc., Deerfield, IL, USA) adsorbent were fixed in place at the apical opening of the glass chamber. Purified air was delivered at a rate of 100 ml/min. Collected volatiles were eluted from the traps into vials using methylene chloride (200 µl), and 10 µl of a 0.84 ng/µl solution of 1‐octanol in methylene chloride was added as an internal standard. Volatiles were analyzed using a gas chromatograph (GC Agilent 6890; Agilent Technologies, Santa Clara, CA, USA) coupled to a mass selective detector (MSD, Agilent 5973; Agilent Technologies). Quantification of compounds was made relative to the internal standard.

### 2.4 Field study

Two locations were selected for field assays. Relative densities of D. carinulata were determined during initial surveys. The first site averaged 10 adults per three 1‐m sweeps, and the second averaged five adults per three 1‐m sweeps. The first site (44°46.489'N: 108°11.113'W) was designated a high‐density site, and the second (44°50.705'N: 108°11.958'W) was designated a low‐density site. Sites were ∼ 8 km apart and located in the greasewood floodplain of the Bighorn River. The greasewood floodplains are characterized by having average Tamarix densities of 0.68 plants/m^2^.^44^ At each location, the existing array of plants was randomly assigned one of four semiochemical treatments. Plants were treated with either BL, PH, PL or PHPL. BL acted as the control treatment in this study and PHPL was a combined treatment of PH and PL. Twelve replicates of each treatment were used in 2013, and eight replicates were used in 2014. Dollops were applied by hand using a 100‐ml syringe loaded with 80 g of SPLAT formulations prepared by ISCA Technologies. An application of 1.25 ml of formulated matrix yielded 1‐g dollops. Formulations were transported to the field on ice in a cooler and were kept chilled while treatments were applied in the field to maintain the correct viscosity of the matrix. Dollops were applied to cattle ear tags individually enclosed within wire mesh cages and attached to the branches of the designated treatment plants (Fig. S1). Cattle ear tags were caged to minimize potential animal encounter with the dollops. Field doses were applied as 1‐g dollops of the BL, PH and PL treatment with the PHPL treatment presented as a 1‐g dollop of PH and a 1‐g dollop of PL. All treatment plants were spaced ∼ 20 m apart to minimize overlap of the volatile semiochemical treatments.^11^, ^12^

Sampling of treated trees began at emergence of the overwintered P adults and ended with the non‐reproductive F2 adults. Plants were sampled weekly by sweep net.^45^ There were 14 weeks of sampling in 2013 (25 June to 20 September) and 15 weeks of sampling in 2014 (16 June to 17 September). After the plants were swept each week, new 1‐g dollops of the assigned treatments were applied. Sweep samples were taken using a canvas sweep net with an opening diameter of 30.5 cm and a depth of 61 cm. Because of the small size of many plants at the research locations, sweeps were limited to three per plant to minimize redundant sampling of the canopy. After the three sweeps, captured D. carinulata adults and larvae were counted. All swept organisms were placed back on the tree to minimize further disturbance of the insect populations. D. carinulata adults were categorized into two groups for subsequent analysis, reproductive and non‐reproductive. The reproductive population was composed of the P adults and F1 adults, whereas the non‐reproductive population was composed of the F2 adults. Determination of adult reproductive status was based on the condition of the ovaries and ovarioles (female) and accessory glands (male) from dissected individuals^23^ collected adjacent to the research locations. Based on specimen dissections, the first non‐reproductive (F2) adults were determined to have emerged during week 10 of monitoring in 2013 (23 August), and week 11 of monitoring in 2014 (29 August).

Visual estimates were used to quantify feeding damage on treatment plants. Damage was rated as the percentage of foliar tissue damaged by D. carinulata and was assessed in increments of 5%.^45^ D. carinulata feeding on Tamarix results in foliage that turns brown, withers and dies. Feeding can girdle the lower portions of the photosynthetic stems, resulting in the desiccation and eventual death of the foliage above the girdle point. Foliage in this condition was also considered as damaged by D. carinulata, even if feeding was minimal above the girdled tissue. Plants with foliage that was entirely brown or that had lost all foliage were classified as 100% damaged. Defoliation that occurred very early in the season was sometimes followed by partial regrowth of the foliage. For those plants, the percentage of regrowth was estimated for the canopy and was added to the percentage of undamaged green foliage.

Annual foliar dieback was determined in early summer for all monitored plants. Dieback was recorded as the percentage of branches that had live foliage the previous year but had no leaf buds when assessed.^46^ Color and flexibility of branches, and the presence or absence of foliage was used to determine when any dieback occurred. Measurements of dieback were taken on 28 June 2013, 26 June 2014 and 29 June 2015. This occurred well after budburst of Tamarix to allow adequate time for new growth. Field observations place the approximate budburst of Tamarix in early May.

Canopy volume was estimated for all monitored trees. To determine canopy volume, measurements of the maximum length, width and height of the canopy were taken.^46^ These measurements were used to determine if canopy volume was increasing, decreasing or remained constant over the study period.

### 2.5 Statistical analysis

Differences according to semiochemical treatment in mean densities recorded for reproductive P and F1 adults, non‐reproductive F2 adults, and larvae were assessed using a repeated measures analysis of variance (ANOVA) with sampling day as the repeated measure and semiochemical formulation applied as the treatment factor.^7^, ^47^ The P and F1 adults were grouped and categorized as reproductive adults in the analysis and the F2 adults were analyzed as non‐reproductive adults. The counts of reproductive adults, non‐reproductive adults, and larvae were transformed using ln(x + 1) in 2013 to better meet the assumptions of normality and homoscedasticity. In 2014, only the counts for non‐reproductive adults swept from treated plants at the high‐density site were ln(x + 1) transformed as the other variables met requisite assumptions for ANOVA.

Damage ratings and canopy volume were analyzed using a repeated measures ANOVA. Sampling day was used as the repeated measure for damage rating, while year was used as the repeated measure for canopy volume. Both variables used the type of semiochemical applied as the treatment factor. Canopy volume was ln(x + 1) transformed to better match the assumptions of ANOVA. Repeated measures ANOVAs used type III error with Satterthwaite approximation for degrees of freedom using the lmer function in the lmerTest package of R statistical software.^48^ Percentage dieback was analyzed using a one‐way ANOVA. Individual comparisons were made using orthogonal contrasts from the ANOVAs. To determine the relationship between days after application and emission of the semiochemicals from the dollops of SPLAT, release rates were analyzed by regression analysis. All analyses were conducted using R software version 3.1.2.

## 3 RESULTS

### 3.1 Release‐rate analysis

Averaged release rates for the five compounds tested are shown in Table 1. The alcohols 2E,4Z‐7:OH (PH) and 3Z‐6:OH (component of PL) were released at average rates of 1919.8 and 250.1 ng h^−1^, respectively, over the first 10 days. The aldehydes 3Z‐6:Ald and 2E‐6:Ald (two components of PL) were released at average rates of 1362.8 and 106.6 ng h^−1^, respectively, over the same time interval. The acetate ester 3Z‐6:OA (component of PL) was released at an average rate of 251.1 ng h^−1^ over the first 10 days. Emission of the pheromone from the 1‐g dollops on day one was equivalent to ∼ 810 adult male D. carinulata.^11^ On day 10, the dollop emission was equivalent to ∼ 25 adult males. By day 31, the dollop emission was equivalent to the pheromone emissions of 10 adult males. The reduction in N on days 5 and 15 was due to equipment malfunction resulting in the loss of three samples. Reduced N on days 25 and 31 was due to dollops detaching from the cattle ear tags to which they were applied.

**Table 1:** Mean ± SE (ng h^−1^) of semiochemicals emitted from 1‐g dollops of field‐aged dollops over a 31‐day period

Day	2E,4Z‐7:OH (N)	3Z‐6:OH (N)	2E‐6:Ald (N)	3Z‐6:Ald (N)	3Z‐6:OAc (N)
1	4214.4 ± 879.26 (8)	663.66 ± 85.29 (8)	3118.06 ± 390.59 (8)	264.04 ± 31.6 (8)	502.56 ± 83.08 (8)
3	2473.52 ± 353.15 (8)	184.53 ± 25.05 (8)	1275.16 ± 169.37 (8)	70.55 ± 8.1 (8)	259.2 ± 38.05 (8)
5	857.67 ± 231.76 (8)	111.97 ± 24.14 (6)	743.16 ± 159.74 (6)	47.32 ± 6.44 (6)	171.58 ± 42.73 (6)
10	133.57 ± 64.76 (8)	40.21 ± 5.12 (8)	314.93 ± 35.49 (8)	44.59 ± 4.57 (8)	71.11 ± 8.55 (8)
15	71.07 ± 17.02 (8)	62.96 ± 24.05 (7)	459.14 ± 185.75 (7)	55.61 ± 9.1 (7)	84.63 ± 22.38 (7)
25	151.27 ± 91.65 (6)	68.23 ± 34.64 (7)	379.14 ± 176.52 (7)	48.53 ± 16.4 (7)	79.68 ± 25.6 (7)
31	53.23 ± 14.17 (5)	49.4 ± 24.13 (7)	284.69 ± 115.18 (7)	23.84 ± 7.75 (7)	62.81 ± 18.77 (7)
Best fit equation	y = 5614x ^−1.35^	y = 509x ^−1.31^	y = 2866x ^−1.22^	y = 193x ^−0.97^	y = 507x ^−1.11^
R ^2^	0.89	0.86	0.93	0.81	0.94

### 3.2 Field study

#### 3.2.1 *High‐density site reproductive adult response*

**Figure 1:**
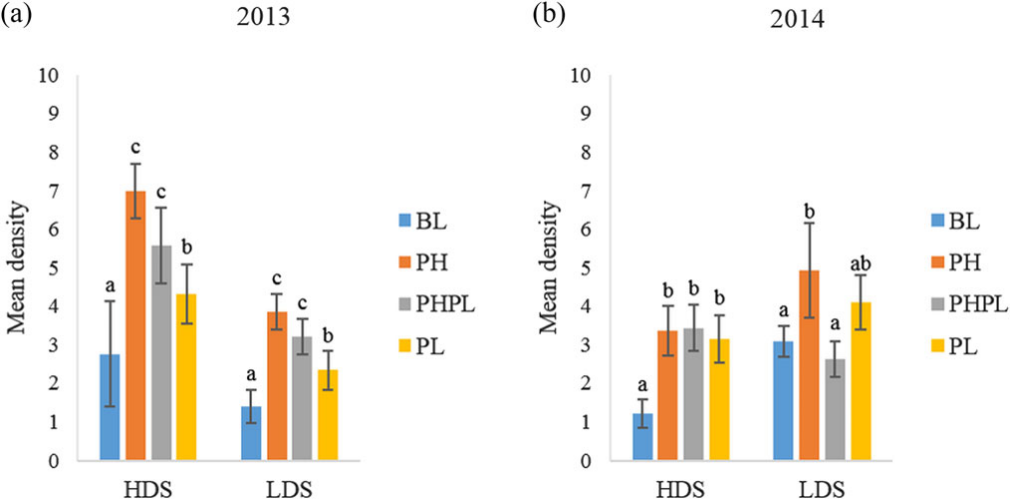
Density expressed as mean ± SE capture of reproductive adult Diorhabda carinulata per three sweeps in (a) 2013 and (b) 2014 at the high‐density (HDS) or low‐density (LDS) site on plants receiving 1‐g dollops of treatment formulation. Treatments applied included blank (BL), pheromone (PH), pheromone and plant volatile (PHPL) or plant volatile (PL). Different letters above error bars denote significant differences between treatments.

In 2013, the number of reproductive adult beetles swept from BL was significantly lower than densities swept from PH, PHPL and PL (PH: P < 0.0001; 3, 420 d.f.; PHPL: P < 0.0001; 3, 420 d.f.; PL: P = 0.014; 3, 420 d.f.) (Fig. 1a). The number of reproductive adults swept from PL was intermediate, with densities higher than swept from BL, but lower than swept from PH (P = 0.01; 3, 419 d.f.) and PHPL (P = 0.009; 3, 419 d.f.). In 2014, higher densities of adult D. carinulata were swept from PH, PHPL and PL than from BL (P = 0.03; 3, 276 d.f.) (Fig. 1b).

#### 3.2.2 *Low‐density site reproductive adult response*

In 2013, the number of reproductive adult beetles swept from BL was significantly lower than densities swept from PH, PHPL and PL (PH: *P* < 0.0001; 3, 420 d.f.; PHPL: *P* < 0.0001; 3, 420 d.f.; PL: *P* = 0.003; 3, 420 d.f.). The number of reproductive adults swept from PL was intermediate, with densities higher than swept from BL, but lower than swept from PH (*P* = 0.0003; 3, 419 d.f.) and PHPL (*P* = 0.045; 3, 419 d.f.). In 2014, only the number of adults swept from PH was greater than those swept from BL (*P* = 0.02; 3, 276 d.f.) (Fig. 1b).

#### 3.2.3 *High‐density site non‐reproductive adult response*

**Figure 2:**
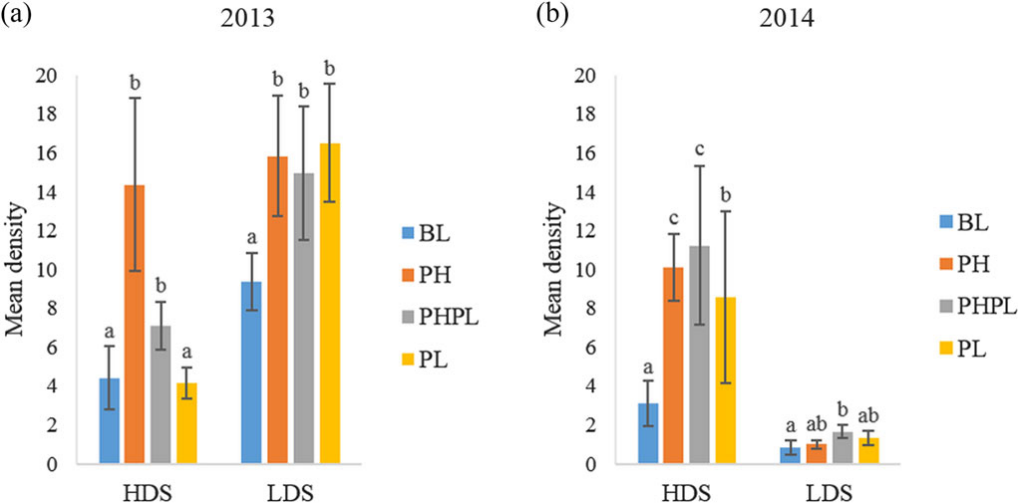
Density expressed as mean ± SE capture of non‐reproductive adult Diorhabda carinulata per three sweeps in (a) 2013 and (b) 2014 at the high‐density (HDS) or low‐density (LDS) site on plants receiving 1‐g dollops of treatment formulation. Treatments applied included blank (BL), pheromone (PH), pheromone and plant volatile (PHPL) and plant volatile (PL). Different letters above error bars denote significant differences between treatments.

In 2013, the number of non‐reproductive adults swept from PH and PHPL was greater than swept from BL (PH: *P* = 0.0001; 3, 233 d.f.; PHPL: *P =* 0.0002; 3, 233 d.f.) (Fig. 2a). In 2014, the number of non‐reproductive adults swept from BL was lower than swept from PH (*P* < 0.0001; 3, 90 d.f.), PHPL (*P* = 0.0001; 3, 90 d.f.), and PL (*P* = 0.04; 3, 90 d.f.) (Fig. 2b). Non‐reproductive adults swept from PL were of intermediate density, higher than BL, but lower than PH (*P* = 0.01; 3, 89 d.f.) and PHPL (*P* = 0.03; 3, 89 d.f.).

#### 3.2.4 *Low‐density site non‐reproductive adult response*

In 2013, the density of non‐reproductive adults swept from BL was lower than from PH, PHPL and PL (PH: *P* = 0.0004; 3, 232 d.f.; PHPL: *P* = 0.003; 3, 232 d.f.; PL: *P* = 0.049; 3, 232 d.f.) (Fig. 2a). In 2014, the density of non‐reproductive adults swept from BL was lower than from the PHPL (*P* = 0.04; 3, 90 d.f.) (Fig. 2b).

#### 3.2.5 *High‐density site larval response*

**Figure 3:**
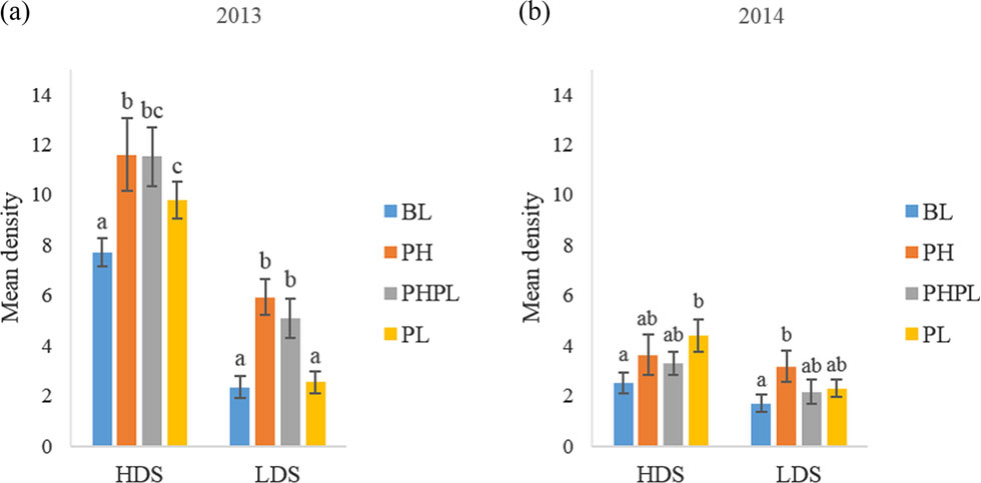
Density expressed as mean ± SE capture of larval Diorhabda carinulata per three sweeps in (a) 2013 and (b) 2014 at the high‐density (HDS) or low‐density (LDS) site on plants receiving 1‐g dollops of treatment formulation. Treatments applied included blank (BL), pheromone (PH), pheromone and plant volatile (PHPL), and plant volatile (PL). Different letters above error bars denote significant differences between treatments.

In 2013, the number of larvae swept from BL was lowest from PH (*P* < 0.0001; 3, 561 d.f.), PHPL (*P* = 0.002; 3, 560 d.f.) and PL (*P* = 0.04; 3, 560 d.f.) (Fig. 3a). There was no difference in the number of larvae swept from PL and PHPL (*P* = 0.28 on 3, 560 d.f.), but was lower than the number of larvae swept from PH (*P* = 0.012 on 3, 560 d.f.). In 2014, the only difference in the number of larvae swept was observed between the BL and PL (*P* = 0.022; 3, 369 d.f.), with PL having the greatest number of larvae swept.

#### 3.2.6 *Low‐density site larval response*

In 2013, the number of larvae swept from BL was significantly lower than from PH (*P* < 0.0001; 3, 561 d.f.) and PHPL (*P* < 0.0001; 3, 561 d.f.) (Fig. 3a). The number of larvae swept from PH and PHPL did not differ (*P* = 0.18; 3, 560 d.f.), but the number swept from both treatments was greater than from PL (PH: *P* < 0.0001; 3, 560 d.f.; PHPL: *P* = 0.003; 3, 560 d.f.). In 2014, few differences in larval densities were observed (Fig. 3b). More larvae were swept from PH than from BL (*P* = 0.004; 3, 369 d.f.) (Fig. 3b).

#### 3.2.7 *High‐density site damage rating*

**Figure 4:**
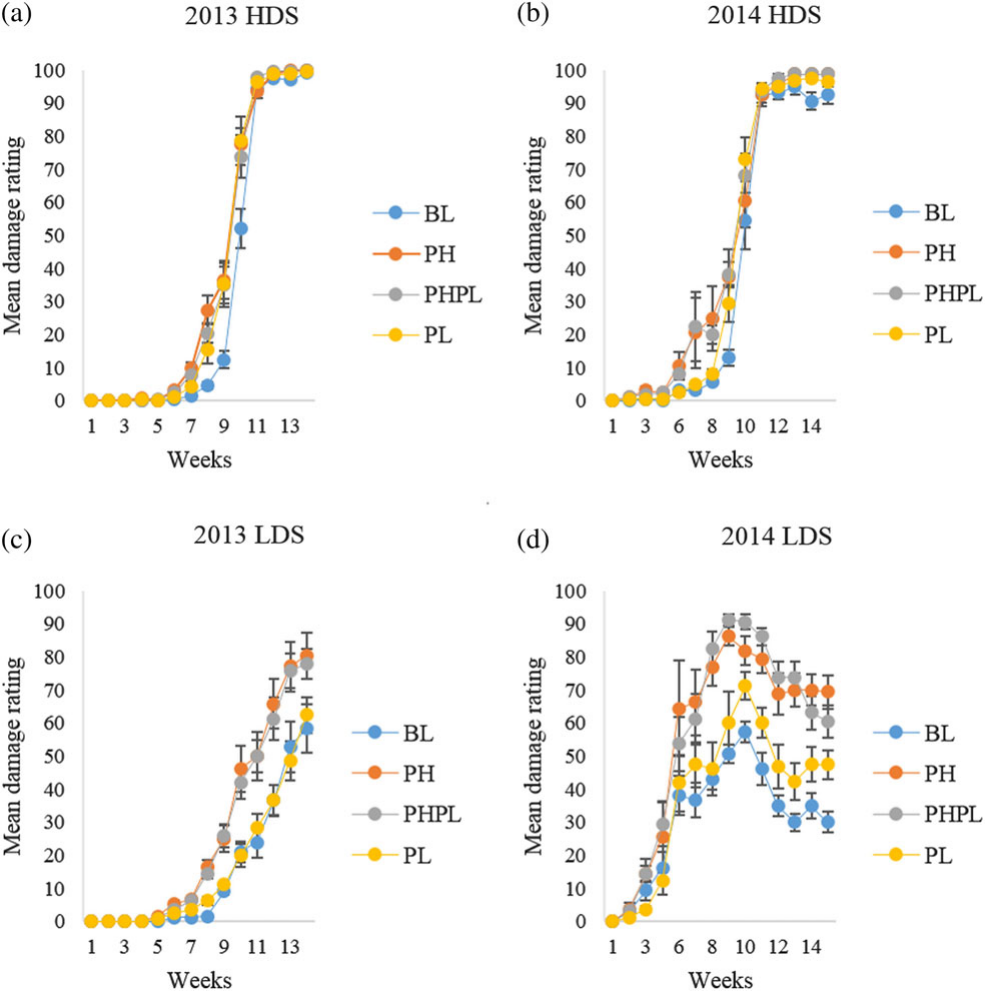
Mean ± SE damage rating for the high‐density site (HDS) in (a) 2013 and (b) 2014, and the low‐density site (LDS) in (c) 2013 and (d) 2014 on plants receiving 1‐g dollops of treatment formulation. Treatments applied included blank (BL), pheromone (PH), pheromone and plant volatile (PHPL) and plant volatile (PL). (a) BL^a^, PH^b^, PHPL^b^, PL^b^; (b) BL^a^, PH^b^, PHPL^b^, PL^c^; (c) BL^a^, PH^b^, PHPL^b^, PL^a^; and (d) BL^a^, PH^b^, PHPL^b^, PL^c^. Different letters next to each treatment denote significant differences between the means.

Greater damage was found throughout the summer on plants treated with the semiochemical formulations than on BL in 2013 (Fig. 4a). The damage evident on BL was lower than on PH (*P* < 0.001; 3, 655 d.f.), PHPL (*P* < 0.001; 3, 655 d.f.) and PL (*P* < 0.001; 3, 655 d.f.). In 2014, damage evident on BL was lower than on PH (*P* < 0.001; 3, 462 d.f.), PHPL (*P* < 0.001; 3, 462 d.f.) and PL (*P* = 0.005; 3, 462 d.f.) (Fig. 4b). Damage evident on PL was lower than on PH (*P* = 0.02; 3, 462 d.f.) and PHPL (*P* = 0.01; 3, 462 d.f.), but higher than on BL.

#### 3.2.8 *Low‐density site damage rating*

In 2013, damage evident on PH and PHPL did not differ (*P* = 0.41; 3, 655 d.f.). Damage on BL in 2013 was lower than on PH (*P* < 0.001; 3, 655 d.f.) and PHPL (*P* < 0.001; 3, 655 d.f.), but did not differ from PL (*P* = 0.49; 3, 655 d.f.) (Fig. 4c). Damage recorded on BL in 2014 was lower than on plants treated with any semiochemical‐containing formulations: PH (*P* < 0.001; 3, 462 d.f.), PHPL (*P* < 0.001; 3, 462 d.f.), and PL (*P* = 0.001; 3, 462 d.f.) (Fig. 4d). Damage on PL in 2014 was also lower than on PH (*P* < 0.001; 3, 462 d.f.) and PHPL (*P* < 0.001; 3, 462 d.f.), but was higher on BL.

#### 3.2.9 *Dieback rating*

**Figure 5:**
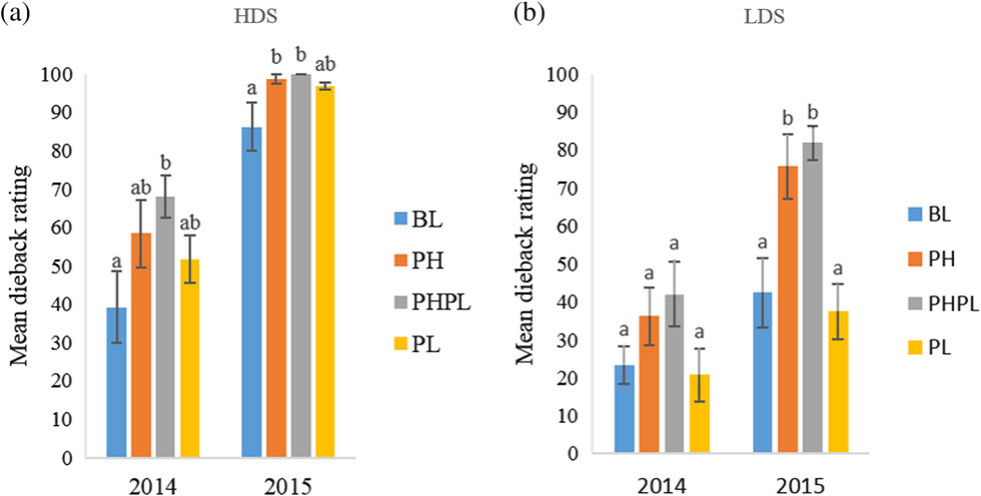
Mean ± SE dieback rating 2014–2015 at (a) the high‐density site (HDS) or (b) the low‐density site (LDS) on plants receiving 1‐g dollops of treatment formulation. Treatments applied included blank (BL), pheromone (PH), pheromone and plant volatile (PHPL) and plant volatile (PL). Different letters above error bars denote significant differences between treatments.

At the high‐density site in 2014, PHPL had greater dieback than the BL (*P* = 0.47; 3, 44 d.f.) (Fig. 5a). At the high‐density site in 2015, both PH (*P* = 0.05; 3, 28 d.f.) and PHPL (*P* = 0.03; 3, 28 d. f.) had greater dieback than BL. At the low‐density site in 2014, dieback on PH, PHPL and PL did not differ from dieback on BL (*P* = 0.12; 3, 44 d.f.) (Fig. 5b). However, in 2015, PH (*P* = 0.02; 3, 28 d.f.) and PHPL (*P* = 0.005; 3, 28 d.f.) sustained significantly greater dieback than BL at the low‐density site.

#### 3.2.10 *Canopy volume measurements*

**Figure 6:**
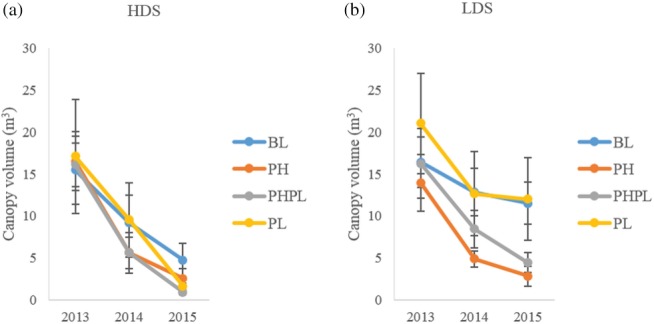
Mean ± SE canopy volume estimated 2013–2015 for (a) the high‐density site (HDS) or (b) the low‐density site (LDS) on plants receiving 1‐g dollops of treatment formulation. Treatments applied included blank (BL), pheromone (PH), pheromone and plant volatile (PHPL) and plant volatile (PL). (a) BL^a^, PH^a^, PHPL^a^, PL^a^; and (b) BL^a^, PH^b^, PHPL^b^, PL^a^. Different letters next to each treatment denote significant differences between the means.

At the high‐density site, no differences in canopy volume were detected for any plant treatments over the 3‐year monitoring period, 2013–2015 (P = 0.49; 3, 90 d.f.) (Fig. 6a). However, at the low‐density site canopy volumes for PH (P < 0.001; 3, 90 d.f.) and PHPL (P < 0.04; 3, 90 d.f.) were smaller than that observed for BL over the 3 years of monitoring (Fig. 6b).

## 4 DISCUSSION

This study elucidated behavioral responses of a weed biological control agent, the northern tamarisk beetle *D. carinulata*, to environmental deployment of two classes of behaviorally active semiochemicals. The purpose of these field applications was to assess the potential enhancement of classical biological control of saltcedar, *Tamarix* spp. The results of our investigation confirmed that previously characterized semiochemicals from *D. carinulata*–*Tamarix* interactions, including both an aggregation pheromone and attractive host‐plant volatile compounds, can intensify herbivory by attracting and retaining greater densities of reproductive and non‐reproductive adults of this weed biological control agent. The result is intensified herbivory with increased foliar damage, dieback and canopy reduction. We believe these results report a novel use of semiochemicals, to attract and retain beneficial defoliating insects and thereby yield greater defoliation of targeted plants for pest management purposes. This effect of increased biological control efficacy could have broad ecological implications, considering *Tamarix* biological control is the method most resilient to secondary invasion of other noxious weeds.^49^, ^50^

Emission rates of the semiochemicals were variable and diminished rapidly after 7 days. After day 7, the much lower release rates stabilized for the next 24 days. Emission of the pheromone from the dollops was equivalent to the amount released by 810 adult males per h on day 1 and decreased to an amount equivalent to the hourly release by a group of 10 adult males by day 31. Although it is difficult to determine whether emission of the pheromone from the dollops after the first 7 days is biologically relevant, the amount emitted is likely to be negligible in the field. When one considers that aggregations of *D. carinulata* can be abundant and contain several hundred individuals, the emissions of 10 males are likely to be insignificant. *Tamarix* plants and female *D. carinulata* also release the aggregation pheromone, although the emissions from females are believed to be derived from the host plant and females have no control over release of the pheormone.^11^ Considering that females and *Tamarix* can also produce the aggregation pheromone, it is likely that high emissions rates from the dollops will be needed to overcome background levels of the pheromone in the environment and facilitate aggregations of *D. carinulata*.

The response of *D. carinulata* to the semiochemical treatments was similar at all our study sites, independent of whether existing populations of *D. carinulata* were at high or low densities. Densities of *D. carinulata* adults were generally highest on *Tamarix* plants treated with the aggregation pheromone. Plants receiving combined aggregation pheromone and plant volatile formulations typically experienced adult densities as high as the aggregation pheromone treatment, but these were sometimes intermediate over the course of the study. Plants receiving the plant volatile formulations were frequently intermediate, with lower adult densities than treatments containing the aggregation pheromone.

Both the pheromone and plant volatile treatments resulted in adult densities that were consistently greater than those on control plants. Regardless of the densities or reproductive status of *D. carinulata*, their spatial distribution can be manipulated through application of the aggregation pheromone. Application of the aggregation pheromone attracted reproductive adults and unexpectedly non‐reproductive adults, even though non‐reproductive males do not produce the aggregation pheromone. Responses to applications of host‐plant volatile treatments may be more dependent on the densities of *D. carinulata* or on site characteristics, because the application of the host‐plant volatiles only periodically increased the densities of *D. carinulata* above the levels seen on the control plants.

Cossé *et al*.^12^ reported a synergistic effect when both aggregation pheromone and behaviorally active green leaf volatiles were used as bait to trap adults. This synergism was not evident in the present study. Densities of reproductive and non‐reproductive adult (Figs 1 and 2, respectively) and larval (Fig. 3) *D. carinulata* swept from plants treated with both the aggregation pheromone and plant volatiles differed only once from densities on plants treated with the pheromone only. In the sole case where these densities differed, the number of reproductive adults swept from plants treated with both the pheromone and plant attractants was significantly lower than the number swept from plants treated with the pheromone only (Fig. 1b).

Increased densities of *D. carinulata* adults and larvae were directly correlated with increased damage to plants, especially in the pheromone and pheromone plus plant volatile treatments (Fig. 4). At many points during the summer, the semiochemical‐treated plants had damage ratings double those of control plants, with some of the greatest differences in damage rating observed in week 8 of monitoring at the high‐density site in 2013 (six times the damage rating of the control). Even though plants at the high‐density site experienced complete defoliation in 2013 and 2014, the greater levels of damage that the semiochemical‐treated plants experienced earlier in the summer resulted in greater levels of dieback in 2014 and 2015. This study demonstrates that damage to a target *Tamarix* plant can be increased through the application of semiochemicals that facilitate or increase the size of *D. carinulata* aggregations. Along with increased foliar damage, an elevated level of *Tamarix* dieback was caused by the application of the semiochemical treatments, especially the aggregation pheromone, whether alone or in combination with the plant volatiles (Figs S2 and S3).

Over the course of this study, canopy volumes of the monitored plants were reduced independent of semiochemical treatment (Fig. 6). It is not surprising that there were no significant differences in canopy volume where *D. carinulata* were present at higher densities, because all monitored plants on the high‐density site were eventually defoliated. This highlights the fact that if enough *D. carinulata* are in the system, the effects of semiochemical manipulation can be diminished. This is because all plants are destined to become defoliated regardless of the treatment. In contrast to the effects seen at higher *D. carinulata* densities, when beetle densities were lower, none of the plants receiving the control treatment were completely defoliated. It was not until the third year of this study that treatment effects became apparent in the plant canopy volume. This supports the idea that plants can recover and have a certain level of tolerance to *D. carinulata* feeding.^51^

However, this tolerance can be overwhelmed by the application of semiochemicals, resulting in a greater than expected reduction in canopy volume. Plants exposed to 2 years of defoliation commonly experience a 25–33% reduction in canopy.^49^ Our control plants exposed to lower beetle densities corroborate these results, with a reduction in plant canopy volume of 30% after 2 years of *D. carinulata* activity. In contrast, the reduction in plant canopy volume in semiochemical‐treated plants was 43% for the plant volatile treatment, 73% for the combined aggregation pheromone and plant volatile treatment, and 79% for the pheromone‐only treatment. These levels of canopy reduction are much higher than expected for the existing *D. carinulata* population density.

Two confounding variables were apparent through this study. *D. carinulata* are commonly known to avoid previously defoliated plants and the larvae are mobile, with the ability to move away from defoliated plants.^18^, ^49^ Because plants treated with the semiochemicals were subjected to more intensive feeding by *D. carinulata* (Fig. 4), there is a possibility that such intensified feeding by *D. carinulata* may have caused physiological changes in these plants, thereby reducing their attractiveness.^49^ It is possible that the response of the plants to semiochemical treatments could cause the control plants and the less successful plant volatile treatment to be more attractive to the *D. carinulata* adults the following year, compared with plants heavily damaged via pheromone and pheromone plus plant volatile treatments. This confounding variable was likely having an impact on the densities of the reproductive and non‐reproductive adults and larvae in 2014 because differing levels of maximum damage rating occurred the previous year (Fig. 4c). Because all the plants experienced near complete defoliation when *D. carinulata* were present at higher densities, these plants are expected to have similar changes in physiology the following year, thus minimizing host preference at this site (Fig. 4a).

At many points during this study, large numbers of third instars were observed on plants that showed no evidence of previous infestation by *D. carinulata*. The cast exoskeletons of the earlier instars are prevalent on the foliage of *Tamarix*, but were not observed in this case. This suggests that the third instars did not develop on the plants on which they were found, but instead migrated to them from adjacent plants. These two confounding variables, the possibility that intensive feeding physiologically alters *Tamarix* to make regrowth less attractive to *D. carinulata*, and the migration of larval beetles between host plants may, over time, have resulted in inflated beetle capture densities on the less successful BL or PL treatments. However, even with these possible confounding variables, significant differences in *D. carinulata* capture densities, damage ratings, dieback and changes in canopy volume could be attributed to the semiochemical treatments. The application of semiochemical formulations, especially those containing the *D. carinulata* aggregation pheromone (2*E*,4*Z*)‐2,4‐heptadien‐1‐ol, conclusively resulted in increased beetle population densities and herbivory.

This study was exploratory and further refinement requires additional research. Future research should be undertaken to determine the effects of these semiochemical treatments on reproduction, and additional experiments would identify how to optimize semiochemical deployment to achieve herbivory with the greatest efficacy. The methods described here require weekly application of the semiochemicals to achieve season‐long manipulation of *D. carinulata*. This frequency is limiting, so further efforts should address achieving greater efficiency when making applications.

## 5 CONCLUSION

Using aggregation‐causing semiochemicals, we were able to manipulate the spatial distribution of *D. carinulata*, and these manipulations resulted in increased densities of the biological control agent on target plants. These increased densities resulted in significantly enhanced damage by *D. carinulata*. This increased damage resulted in elevated dieback of the plants and a massive reduction in canopy volume in as few as two consecutive years of defoliation. These results of increased damage, dieback and canopy reduction are desirable outcomes for weed managers. Classical biological control of weeds is not intended to yield eradication, rather, the goal is to suppress a weed below ecologically damaging levels. This study clearly demonstrates the potential of semiochemical formulations to increase the efficacy of biological control agents of weeds.

## Supporting information


**Figure S1:** Cattle ear tag caged with wire mesh to support and protect dollops.

**Figure S2:** Pheromone treated plant at the low density site showing the resulting dieback after one defoliation event. Top: plant with 100% damage, photo taken on August 23, 2013. Bottom: same plant with corresponding dieback (80%), photo taken on June 16, 2014

**Figure S3:** Difference in dieback between a plant treated with the pheromone and plant volatiles (PHPL) (left side) compared to an adjacent plant treated with just the plant volatiles (PL) (right side). The pheromone and plant volatile treated plant experienced 80% defoliation in 2013 and 80% dieback in 2014. The plant volatile treated plant experienced 35% defoliation in 2013 and 5% dieback in 2014. Photo taken June 23, 2014 at low density site.

**Figure S4:** Mean ± SE of (2E, 4Z)‐2,4‐heptadien‐1‐ol emitted from 1g dollops. Best fit equation and R^2^ displayed in table 1

**Figure S5:** Mean ± SE of plant volatiles emitted from 1g dollops. Best fit equation and R^2^ displayed in table 1

**Supplementary Table 1:** Mean ± SE damage rating (%) for the high density site (HDS) in 2013 and 2014, and the low density site (LDS) in 2013 and 2014. Treatments included blank (BL), pheromone (PH), pheromone and plant volatile (PHPL), and plant volatile (PL)
