# Supplementary figures and images for: Semiochemicals to enhance herbivory by Diorhabda carinulata aggregations in saltcedar (Tamarix spp.) infestations
Source: Pest Manag Sci. 2018 Feb 23;74(6):1494–503. doi: 10.1002/ps.4848 (PMC5969109; doi:10.1002/ps.4848)

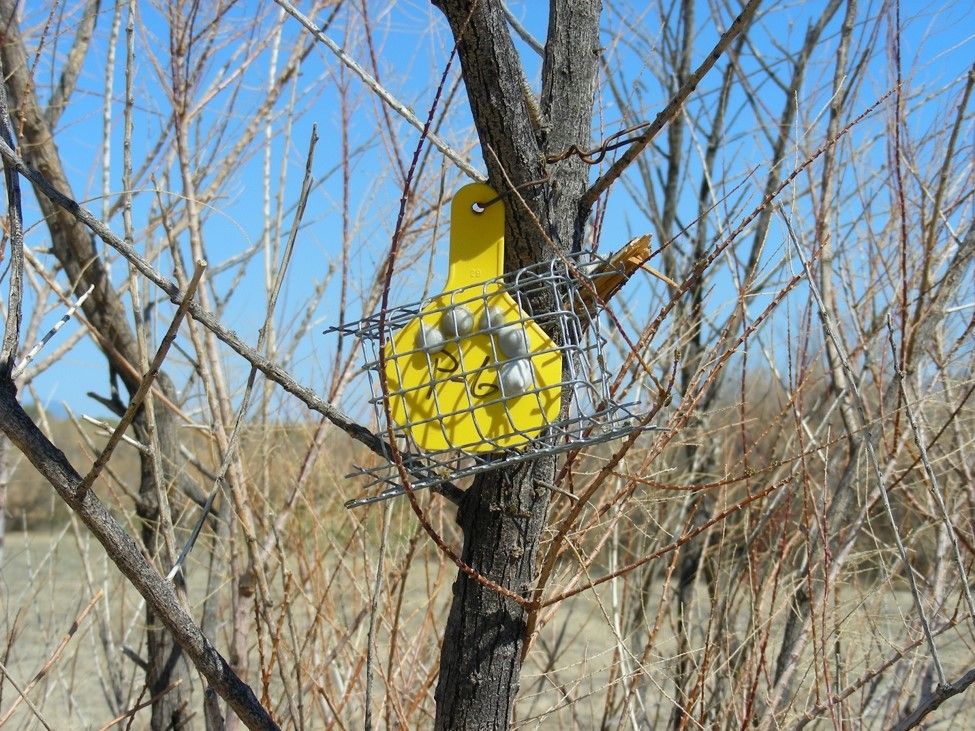

Supplement: Supplementary file 1 — Figure S1: Cattle ear tag caged with wire mesh to support and protect dollops. [file PS-74-1494-s005.jpg]

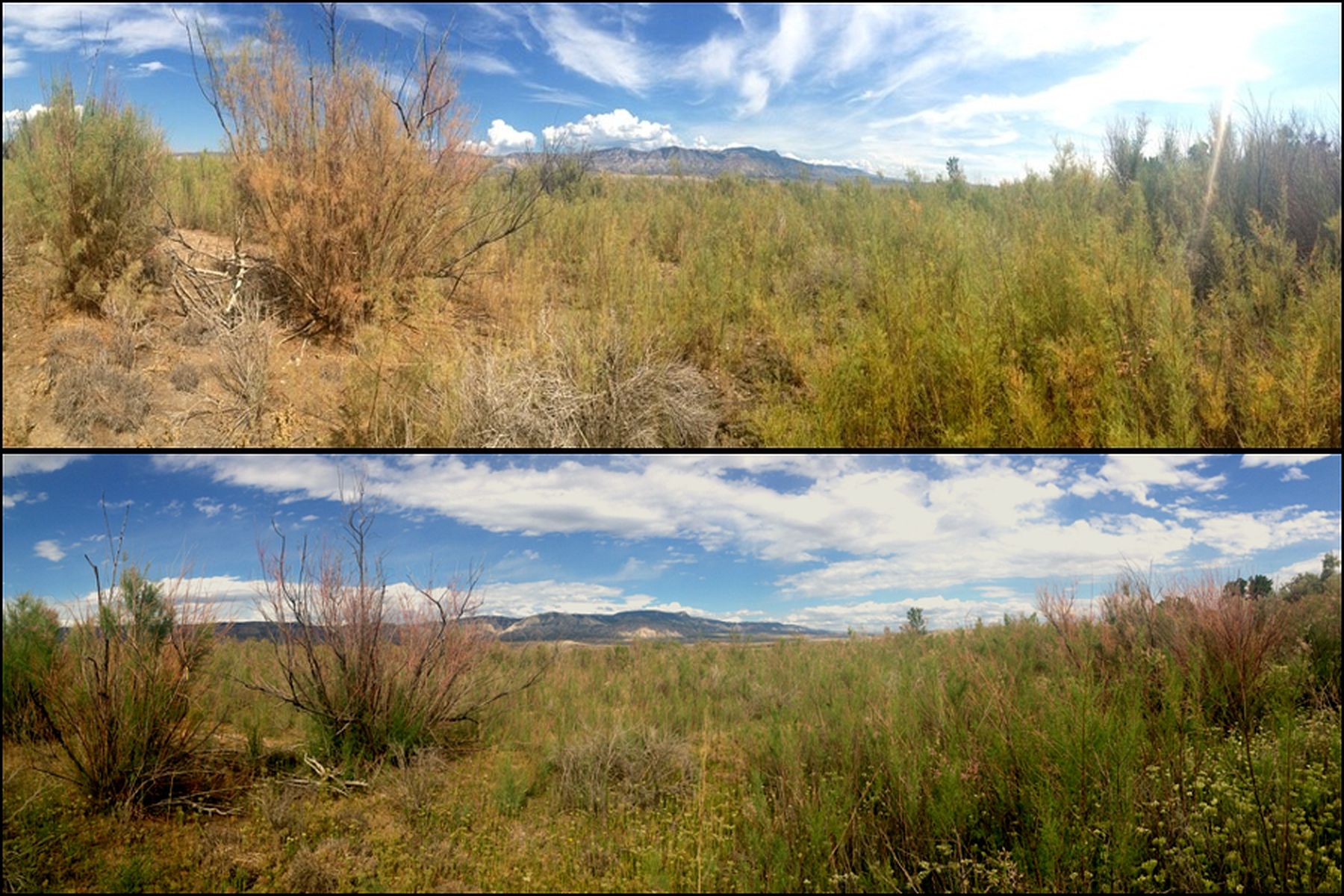

Supplement: Supplementary file 2 — Figure S2: Pheromone treated plant at the low density site showing the resulting dieback after one defoliation event. Top: plant with 100% damage, photo taken on August 23, 2013. Bottom: same plant with corresponding dieback (80%), photo taken on June 16, 2014 [file PS-74-1494-s003.jpg]

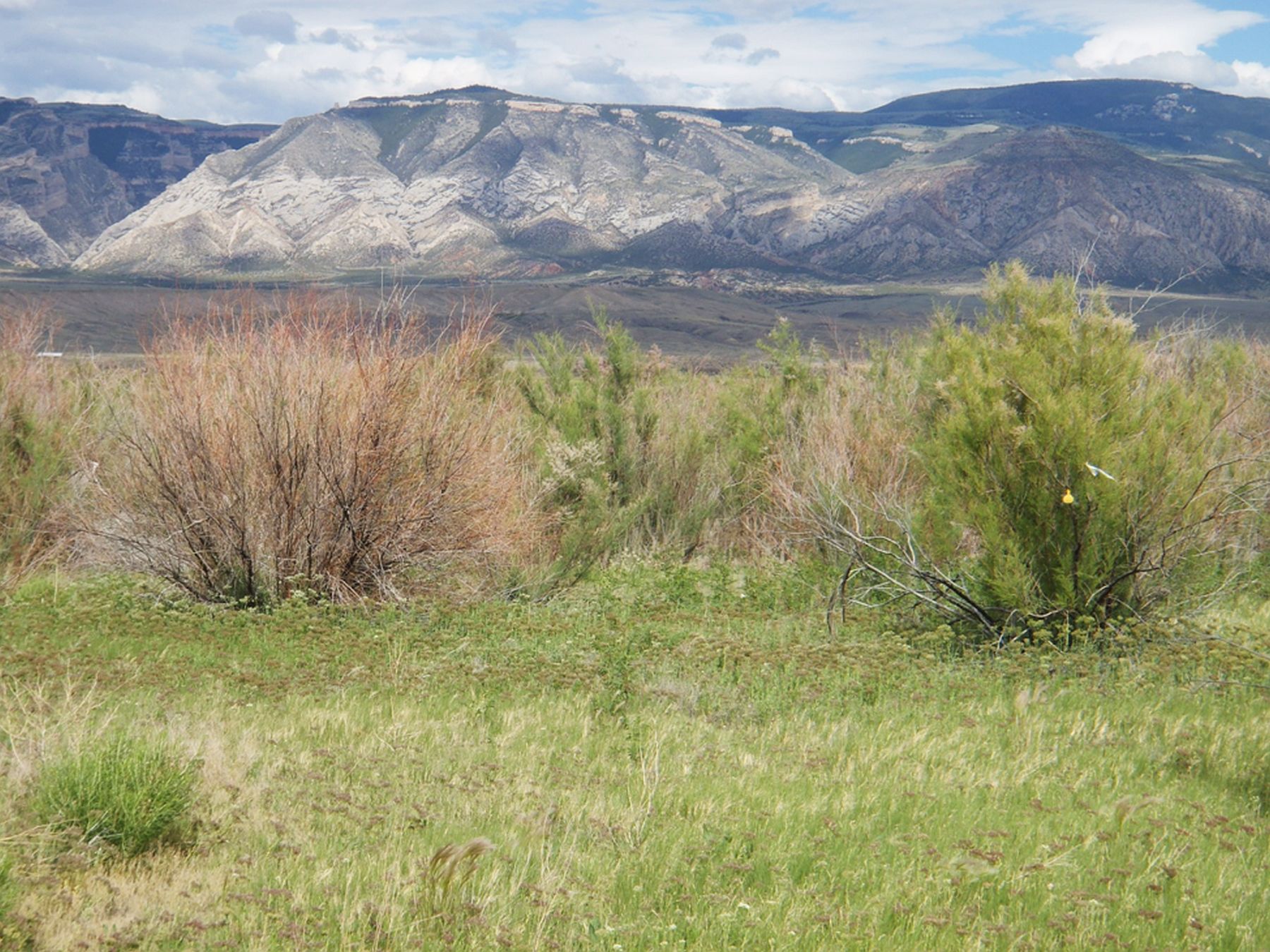

Supplement: Supplementary file 3 — Figure S3: Difference in dieback between a plant treated with the pheromone and plant volatiles (PHPL) (left side) compared to an adjacent plant treated with just the plant volatiles (PL) (right side). The pheromone and plant volatile treated plant experienced 80% defoliation in 2013 and 80% dieback in 2014. The plant volatile treated plant experienced 35% defoliation in 2013 and 5% dieback in 2014. Photo taken June 23, 2014 at low density site. [file PS-74-1494-s001.jpg]

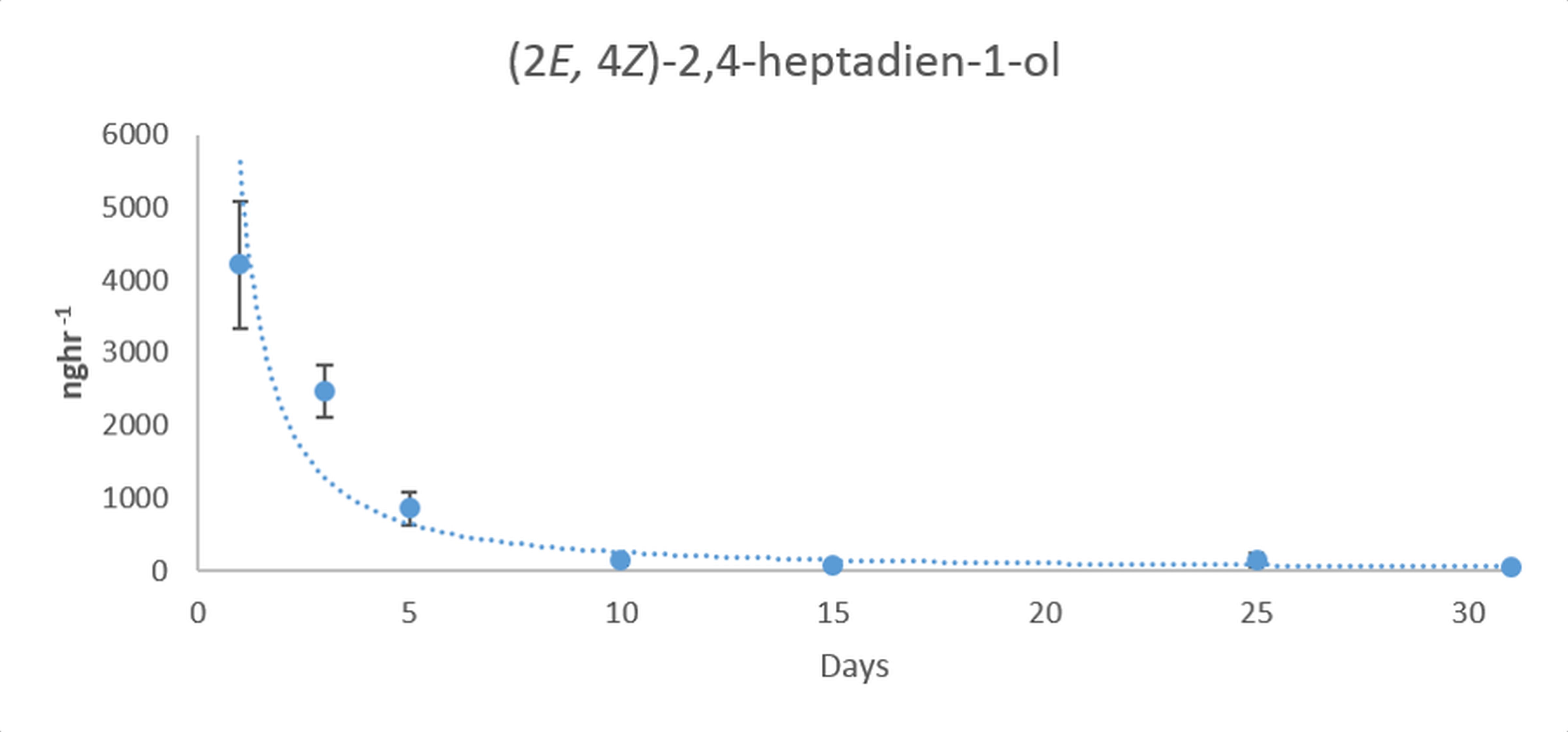

Supplement: Supplementary file 4 — Figure S4: Mean ± SE of (2E, 4Z)‐2,4‐heptadien‐1‐ol emitted from 1g dollops. Best fit equation and R2 displayed in table 1 [file PS-74-1494-s004.jpg]

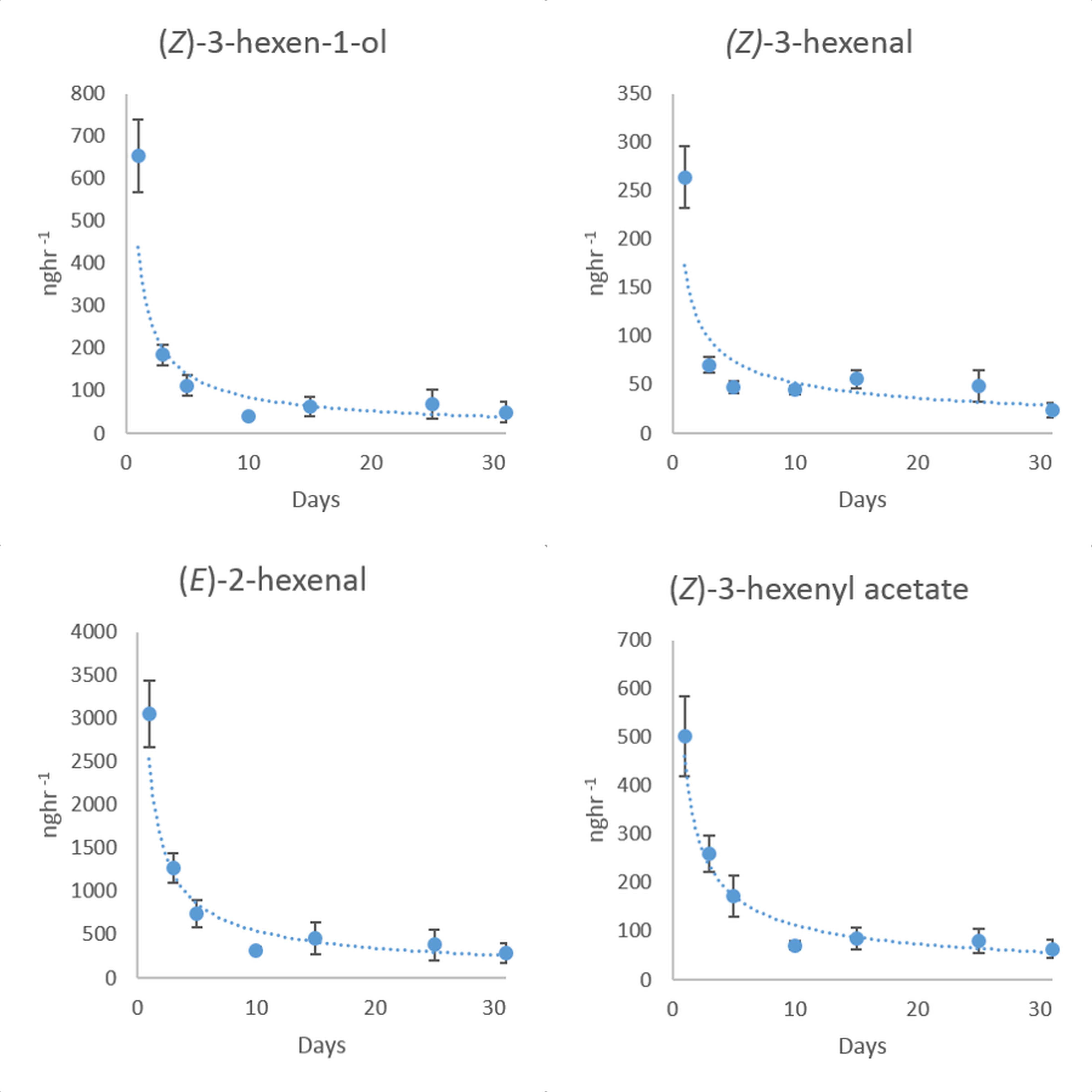

Supplement: Supplementary file 5 — Figure S5: Mean ± SE of plant volatiles emitted from 1g dollops. Best fit equation and R2 displayed in table 1 [file PS-74-1494-s006.jpg]
